# Supplementary material for: DNA Binding and Cleavage, Stopped-Flow Kinetic, Mechanistic, and Molecular Docking Studies of Cationic Ruthenium(II) Nitrosyl Complexes Containing “NS4” Core
Source: Molecules. 2023 Mar 28;28(7):3028. doi: 10.3390/molecules28073028 (PMC10095794; doi:10.3390/molecules28073028)
Supplement: Supplementary file 1 [file molecules-28-03028-s001.zip › molecules-2197253-supplementary.pdf]

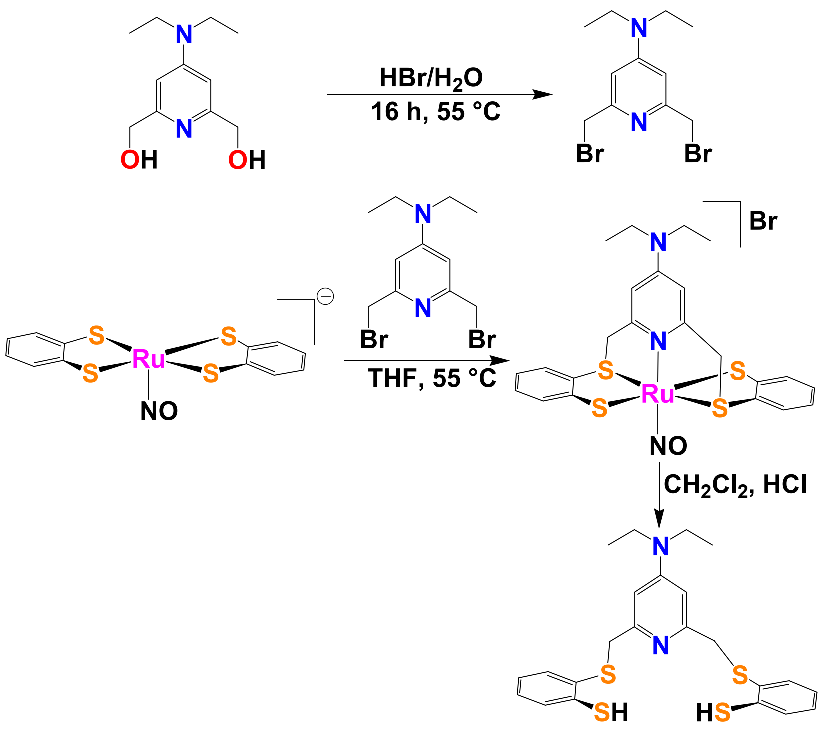

Scheme S1: Synthetic route for [RuNOTSP]Br and its ligand $TSPH_2$, respectively

Geometrical optimization:

Molecular geometry was optimized at the B3LYP level of theory using the bases 6-31 g(d) for **$TSPH_2$** and **$TSPH_2$** and LAN2DZ for metal in **[RuNOTSP]**$^+$. Additional parameters such as HOMO–LUMO energy gap E_g, absolute electronegativities χ, chemical potentials, π, absolute hardness, η, absolute softness, σ, global electrophilicity, ω, global softness, S, and additional electronic charge, ΔN_{max}, can be defined using Koopman's theorem by the following equations:

$$E_g = E_{LUMO} - E_{HOMO}$$

$$\chi = -(E_{HOMO} + E_{LUMO}) / 2$$

$$\eta = (E_{LUMO} - E_{HOMO}) / 2$$

$$\sigma = 1/\eta$$

$$\pi = -\chi$$

$$S = 1/2\eta$$

$$\omega = \pi^2 / 2\eta$$

$$\Delta N_{max} = \pi/\eta$$

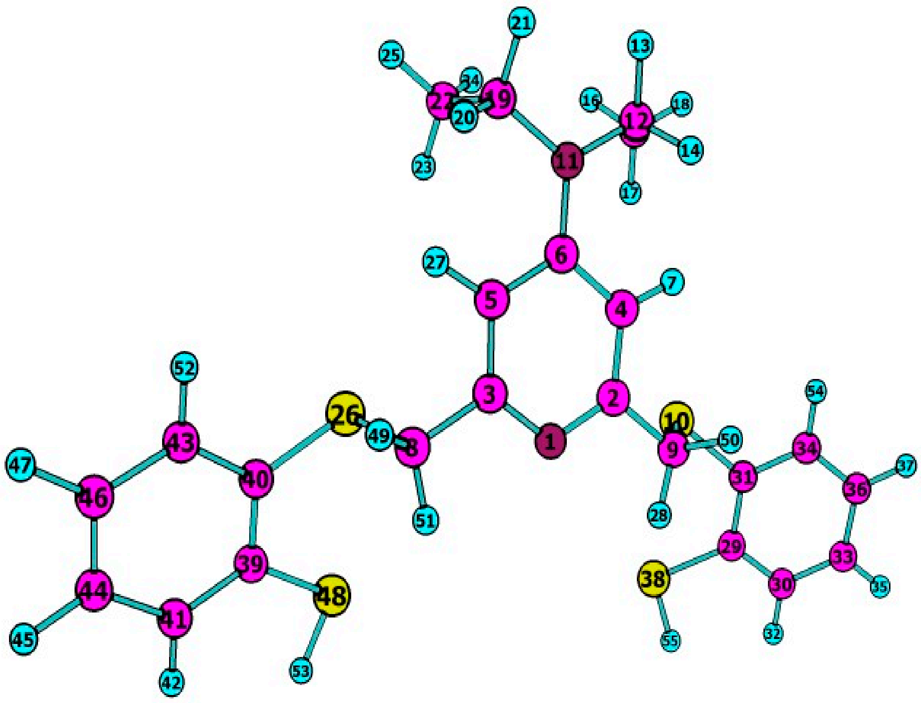

Figure S1: Structure of $TSPH_2$

Table S1. Summary of Natural Population Analysis (NPA) of $TSPH_2$:

Atom	No	Charge	Core	Valence	Rydberg	Total
N	1	-0.48666	1.99937	5.46670	0.02059	7.48666
C	2	0.21078	1.99908	3.76504	0.02511	5.78922
C	3	0.21077	1.99908	3.76505	0.02511	5.78923
C	4	-0.32176	1.99896	4.31209	0.01071	6.32176
C	5	-0.32175	1.99896	4.31207	0.01071	6.32175
C	6	0.21820	1.99905	3.76378	0.01897	5.78180
H	7	0.23970	0.00000	0.75905	0.00125	0.76030
C	8	-0.58698	1.99918	4.57423	0.01357	6.58698
C	9	-0.58699	1.99918	4.57424	0.01356	6.58699
S	10	0.23735	9.99925	5.72735	0.03605	15.76265
N	11	-0.44178	1.99931	5.42986	0.01261	7.44178
C	12	-0.25490	1.99935	4.24022	0.01532	6.25490
H	13	0.23355	0.00000	0.76510	0.00134	0.76645
H	14	0.23170	0.00000	0.76642	0.00188	0.76830
C	15	-0.69017	1.99945	4.68354	0.00719	6.69017
H	16	0.22832	0.00000	0.77069	0.00099	0.77168
H	17	0.24164	0.00000	0.75747	0.00088	0.75836
H	18	0.23416	0.00000	0.76482	0.00102	0.76584
C	19	-0.25490	1.99935	4.24022	0.01532	6.25490
H	20	0.23170	0.00000	0.76642	0.00188	0.76830
H	21	0.23355	0.00000	0.76510	0.00134	0.76645
C	22	-0.69018	1.99945	4.68354	0.00719	6.69018
H	23	0.24164	0.00000	0.75747	0.00088	0.75836

H	24	0.22832	0.00000	0.77069	0.00099	0.77168
H	25	0.23416	0.00000	0.76483	0.00102	0.76584
S	26	0.23736	9.99925	5.72734	0.03605	15.76264
H	27	0.23968	0.00000	0.75906	0.00125	0.76032
H	28	0.27205	0.00000	0.72522	0.00273	0.72795
C	29	-0.17184	1.99866	4.15338	0.01981	6.17184
C	30	-0.25019	1.99897	4.23683	0.01439	6.25019
C	31	-0.22673	1.99867	4.20409	0.02398	6.22673
H	32	0.23697	0.00000	0.76208	0.00095	0.76303
C	33	-0.21371	1.99909	4.20086	0.01376	6.21371
C	34	-0.21224	1.99898	4.19739	0.01588	6.21224
H	35	0.24004	0.00000	0.75912	0.00084	0.75996
C	36	-0.24770	1.99908	4.23483	0.01379	6.24770
H	37	0.23949	0.00000	0.75967	0.00084	0.76051
S	38	0.06633	9.99927	5.89503	0.03937	15.93367
C	39	-0.17184	1.99866	4.15338	0.01981	6.17184
C	40	-0.22673	1.99867	4.20409	0.02398	6.22673
C	41	-0.25019	1.99897	4.23683	0.01439	6.25019
H	42	0.23697	0.00000	0.76208	0.00095	0.76303
C	43	-0.21224	1.99898	4.19739	0.01588	6.21224
C	44	-0.21372	1.99909	4.20086	0.01376	6.21372
H	45	0.24004	0.00000	0.75912	0.00084	0.75996
C	46	-0.24770	1.99908	4.23483	0.01379	6.24770
H	47	0.23949	0.00000	0.75967	0.00084	0.76051
S	48	0.06634	9.99927	5.89503	0.03937	15.93366
H	49	0.24802	0.00000	0.74966	0.00232	0.75198
H	50	0.24803	0.00000	0.74965	0.00232	0.75197
H	51	0.27205	0.00000	0.72522	0.00273	0.72795
H	52	0.24670	0.00000	0.75210	0.00120	0.75330
H	53	0.12455	0.00000	0.87445	0.00100	0.87545
H	54	0.24670	0.00000	0.75210	0.00120	0.75330
H	55	0.12455	0.00000	0.87445	0.00100	0.87545

Table S2. Coordinates parameters for $TSPH_2$:

Center Number	Coordinates (Angstroms) X	Y	Z
1	0.000132	0.028158	-0.953795
2	-1.141835	0.725110	-0.846700
3	1.142019	0.725260	-0.846798
4	-1.195801	2.099246	-0.622226
5	1.195824	2.099400	-0.622320
6	-0.000031	2.844720	-0.503011
7	-2.166618	2.568401	-0.526723
8	2.415563	-0.076527	-0.985819
9	-2.415267	-0.076875	-0.985712
10	3.341532	-0.089022	0.630747
11	-0.000105	4.210097	-0.305411
12	-1.254761	4.943675	-0.133275
13	-1.048427	5.992638	-0.370175
14	-1.972247	4.601033	-0.888048
15	-1.878776	4.847226	1.266045
16	-1.214258	5.272509	2.025125
17	-2.084726	3.808460	1.541657
18	-2.824780	5.401021	1.294411
19	1.254487	4.943818	-0.133423
20	1.971926	4.601243	-0.888269
21	1.048013	5.992756	-0.370316
22	1.878664	4.847449	1.265831
23	2.084740	3.808705	1.541433
24	1.214188	5.272678	2.024978
25	2.824618	5.401336	1.294089
26	3.341079	-0.089701	0.631078
27	2.166586	2.568682	-0.526873
28	-2.159989	-1.101515	-1.258809
29	-4.858603	-2.402458	0.214595
30	-6.042783	-3.067161	-0.137559
31	-4.818157	-0.993453	0.159114
32	-6.086804	-4.152102	-0.097920
33	-7.169449	-2.348880	-0.531822
34	-5.963705	-0.288002	-0.231878
35	-8.076497	-2.885223	-0.797782
36	-7.137339	-0.954536	-0.578863
37	-8.016384	-0.392210	-0.880218
38	-3.399128	-3.287168	0.740364
39	4.858680	-2.402617	0.213926
40	4.818053	-0.993579	0.159468
41	6.043113	-3.066890	-0.138188
42	6.087270	-4.151854	-0.099341
43	5.963676	-0.287672	-0.230484
44	7.169854	-2.348154	-0.531410
45	8.077099	-2.884167	-0.797363
46	7.137564	-0.953780	-0.577432
47	8.016668	-0.391105	-0.877963

48	3.399097	-3.287924	0.738389
49	3.080786	0.354962	-1.740187
50	-3.080167	0.354021	-1.740710
51	2.160519	-1.101012	-1.259714
52	5.920353	0.797197	-0.255325
53	3.915251	-4.516250	0.510900
54	-5.920529	0.796855	-0.257497
55	-3.914970	-4.515739	0.513485

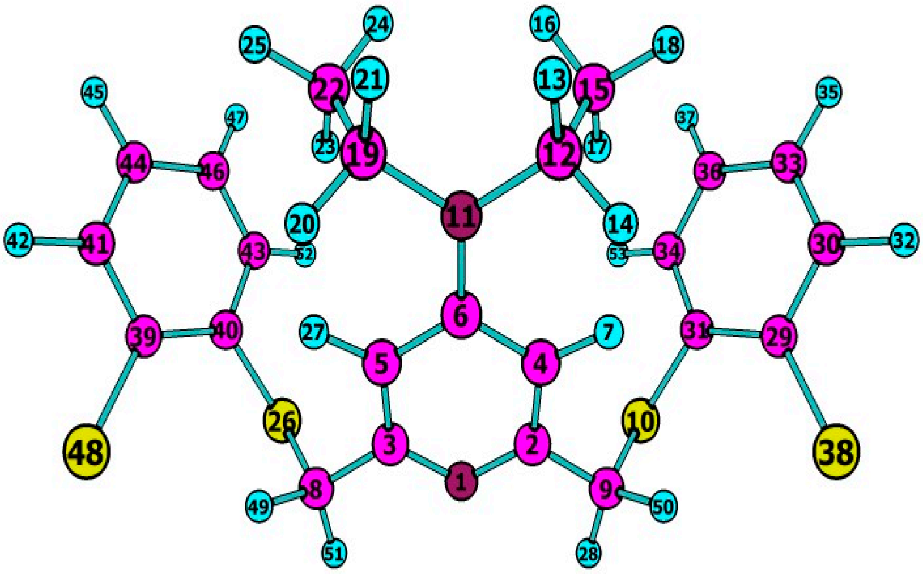

Figure S2: Structure of TSP^{2-}

Table S3. Summary of Natural Population Analysis (NPA) for TSP^{2-} anion.

Atom	No	Charge	Core	Valence	Rydberg	Total
N	1	-0.51020	1.99938	5.49090	0.01993	7.51020
C	2	0.21981	1.99907	3.75563	0.02550	5.78019
C	3	0.21981	1.99907	3.75563	0.02550	5.78019
C	4	-0.33076	1.99896	4.32084	0.01096	6.33076
C	5	-0.33076	1.99896	4.32084	0.01096	6.33076
C	6	0.20540	1.99904	3.77673	0.01884	5.79460
H	7	0.24504	0.00000	0.75365	0.00131	0.75496
C	8	-0.60582	1.99914	4.59162	0.01506	6.60582
C	9	-0.60581	1.99914	4.59162	0.01505	6.60581
S	10	0.21314	9.99924	5.74910	0.03852	15.78686
N	11	-0.45570	1.99931	5.44383	0.01255	7.45570
C	12	-0.24959	1.99936	4.23498	0.01526	6.24959
H	13	0.21220	0.00000	0.78618	0.00161	0.78780
H	14	0.23670	0.00000	0.76125	0.00205	0.76330
C	15	-0.68687	1.99944	4.68002	0.00741	6.68687
H	16	0.21019	0.00000	0.78863	0.00118	0.78981
H	17	0.25782	0.00000	0.74108	0.00110	0.74218
H	18	0.22478	0.00000	0.77390	0.00132	0.77522
C	19	-0.24960	1.99936	4.23499	0.01526	6.24960
H	20	0.23667	0.00000	0.76128	0.00205	0.76333
H	21	0.21222	0.00000	0.78617	0.00161	0.78778
C	22	-0.68686	1.99944	4.68001	0.00741	6.68686
H	23	0.25781	0.00000	0.74109	0.00110	0.74219
H	24	0.21021	0.00000	0.78862	0.00118	0.78979
H	25	0.22479	0.00000	0.77389	0.00132	0.77521
S	26	0.21314	9.99924	5.74910	0.03852	15.78686

H	27	0.24503	0.00000	0.75366	0.00131	0.75497
H	28	0.25233	0.00000	0.74538	0.00229	0.74767
C	29	-0.18021	1.99878	4.15644	0.02500	6.18021
C	30	-0.25823	1.99899	4.24285	0.01639	6.25823
C	31	-0.24501	1.99868	4.22439	0.02194	6.24501
H	32	0.22396	0.00000	0.77413	0.00192	0.77604
C	33	-0.25442	1.99906	4.24187	0.01348	6.25442
C	34	-0.23923	1.99899	4.22460	0.01565	6.23923
H	35	0.20342	0.00000	0.79528	0.00129	0.79658
C	36	-0.30913	1.99906	4.29554	0.01452	6.30913
H	37	0.20803	0.00000	0.79090	0.00107	0.79197
S	38	-0.47654	9.99943	6.45118	0.02593	16.47654
C	39	-0.18021	1.99878	4.15644	0.02500	6.18021
C	40	-0.24500	1.99868	4.22438	0.02194	6.24500
C	41	-0.25823	1.99899	4.24284	0.01639	6.25823
H	42	0.22395	0.00000	0.77413	0.00192	0.77605
C	43	-0.23925	1.99899	4.22461	0.01565	6.23925
C	44	-0.25442	1.99906	4.24188	0.01348	6.25442
H	45	0.20342	0.00000	0.79529	0.00129	0.79658
C	46	-0.30911	1.99906	4.29553	0.01452	6.30911
H	47	0.20803	0.00000	0.79090	0.00107	0.79197
S	48	-0.47655	9.99943	6.45119	0.02593	16.47655
H	49	0.27682	0.00000	0.71958	0.00360	0.72318
H	50	0.27682	0.00000	0.71958	0.00360	0.72318
H	51	0.25233	0.00000	0.74538	0.00229	0.74767
H	52	0.23183	0.00000	0.76675	0.00142	0.76817
H	53	0.23183	0.00000	0.76675	0.00142	0.76817

===

Table S4. Coordinates parameters for TSP^{2-} anion:

Center Number	Coordinates (Angstroms) X	Y	Z
1	0.000007	-2.206649	-0.478068
2	-1.148604	-1.522143	-0.634309
3	1.148520	-1.521993	-0.634396
4	-1.198240	-0.162640	-0.949640
5	1.197962	-0.162481	-0.949703
6	-0.000193	0.560843	-1.128517
7	-2.170811	0.301091	-1.052241
8	2.427777	-2.308862	-0.504302
9	-2.427745	-2.309178	-0.504067
10	-3.346102	-2.111806	1.097816
11	-0.000280	1.900941	-1.503182
12	-1.252039	2.634608	-1.656012
13	-1.049094	3.481791	-2.324079
14	-1.975760	2.000338	-2.180715
15	-1.878547	3.150008	-0.352205
16	-1.206589	3.851228	0.156629
17	-2.103777	2.328876	0.333622
18	-2.822996	3.666526	-0.564315
19	1.251357	2.635100	-1.654774
20	1.975544	2.001562	-2.179741
21	1.048443	3.482842	-2.322125
22	1.877087	3.149447	-0.350183
23	2.102173	2.327727	0.334980
24	1.204692	3.850015	0.158968
25	2.821524	3.666369	-0.561343
26	3.346245	-2.111549	1.097521
27	2.170468	0.301353	-1.052453
28	-2.186930	-3.375107	-0.540125
29	-5.188457	-0.213100	0.014199
30	-5.808158	1.061405	0.178330
31	-4.178994	-0.525639	0.987828
32	-6.573931	1.326757	-0.546722
33	-5.495077	1.939964	1.205949
34	-3.857150	0.383965	2.010132
35	-6.019604	2.894060	1.275343
36	-4.509899	1.608546	2.145971
37	-4.244608	2.290776	2.951358
38	-5.684924	-1.244096	-1.303409
39	5.188726	-0.212990	0.013810
40	4.179336	-0.525486	0.987528
41	5.808591	1.061430	0.177966
42	6.574292	1.326759	-0.547171
43	3.857760	0.384056	2.009972
44	5.495773	1.939925	1.205720
45	6.020432	2.893947	1.275136
46	4.510696	1.608531	2.145854

47	4.245604	2.290712	2.951349
48	5.684922	-1.243930	-1.303947
49	3.154463	-2.072519	-1.289090
50	-3.154531	-2.073017	-1.288818
51	2.187100	-3.374822	-0.540425
52	3.073853	0.107509	2.711026
53	-3.073185	0.107386	2.711106

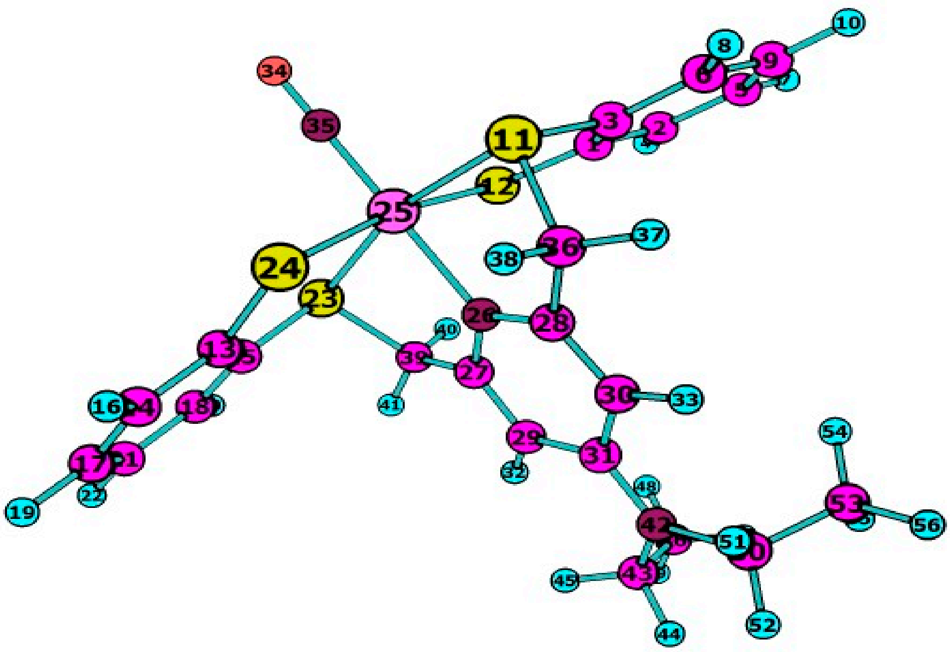

Figure S3: Structure of [RuNOTSP]$^{+}$ cation.

Table S5. Summary of Natural Population Analysis (NPA) of [RuNOTSP]$^{+}$:

Atom	No	Charge	Core	Valence	Rydberg	Total
C	1	-0.18382	1.99853	4.16490	0.02039	6.18382
C	2	-0.22147	1.99884	4.20636	0.01627	6.22147
C	3	-0.25546	1.99850	4.23616	0.02079	6.25546
H	4	0.25619	0.00000	0.74257	0.00124	0.74381
C	5	-0.19494	1.99892	4.18172	0.01430	6.19494
C	6	-0.21998	1.99881	4.20479	0.01638	6.21998
H	7	0.25474	0.00000	0.74432	0.00094	0.74526
H	8	0.25093	0.00000	0.74784	0.00123	0.74907
C	9	-0.23233	1.99891	4.21858	0.01483	6.23233
H	10	0.25433	0.00000	0.74477	0.00090	0.74567
S	11	0.68887	9.99853	5.26937	0.04322	15.31113
S	12	0.12102	9.99886	5.85063	0.02950	15.87898
C	13	-0.18506	1.99853	4.16570	0.02083	6.18506
C	14	-0.21977	1.99884	4.20465	0.01628	6.21977
C	15	-0.25187	1.99850	4.23279	0.02058	6.25187
H	16	0.25695	0.00000	0.74182	0.00124	0.74305
C	17	-0.19507	1.99892	4.18184	0.01431	6.19507
C	18	-0.22051	1.99881	4.20540	0.01630	6.22051
H	19	0.25520	0.00000	0.74387	0.00094	0.74480
H	20	0.24987	0.00000	0.74889	0.00124	0.75013
C	21	-0.22987	1.99892	4.21613	0.01482	6.22987
H	22	0.25459	0.00000	0.74451	0.00090	0.74541
S	23	0.69449	9.99851	5.26383	0.04317	15.30551
S	24	0.15628	9.99880	5.81450	0.03041	15.84372
Ru	25	-0.67395	35.97393	8.66516	0.03485	44.67395
N	26	-0.43862	1.99913	5.41815	0.02135	7.43862
C	27	0.26107	1.99888	3.71942	0.02063	5.73893

C	28	0.26071	1.99888	3.71980	0.02061	5.73929
C	29	-0.30428	1.99880	4.29411	0.01137	6.30428
C	30	-0.30443	1.99880	4.29424	0.01138	6.30443
C	31	0.26035	1.99893	3.72155	0.01916	5.73965
H	32	0.25520	0.00000	0.74379	0.00101	0.74480
H	33	0.25518	0.00000	0.74381	0.00102	0.74482
O	34	-0.14870	1.99974	6.13336	0.01560	8.14870
N	35	0.45495	1.99883	4.49840	0.04783	6.54505
C	36	-0.61742	1.99907	4.60391	0.01445	6.61742
H	37	0.28214	0.00000	0.71625	0.00161	0.71786
H	38	0.29928	0.00000	0.69923	0.00149	0.70072
C	39	-0.61769	1.99907	4.60427	0.01434	6.61769
H	40	0.29868	0.00000	0.69981	0.00150	0.70132
H	41	0.28194	0.00000	0.71647	0.00159	0.71806
N	42	-0.40769	1.99914	5.39593	0.01262	7.40769
C	43	-0.25816	1.99922	4.24379	0.01516	6.25816
H	44	0.25003	0.00000	0.74864	0.00133	0.74997
H	45	0.23565	0.00000	0.76258	0.00177	0.76435
C	46	-0.68342	1.99929	4.67662	0.00752	6.68342
H	47	0.23707	0.00000	0.76186	0.00107	0.76293
H	48	0.23006	0.00000	0.76893	0.00101	0.76994
H	49	0.24874	0.00000	0.75026	0.00100	0.75126
C	50	-0.25819	1.99922	4.24380	0.01517	6.25819
H	51	0.23606	0.00000	0.76218	0.00176	0.76394
H	52	0.25005	0.00000	0.74861	0.00134	0.74995
C	53	-0.68344	1.99929	4.67665	0.00750	6.68344
H	54	0.22983	0.00000	0.76915	0.00101	0.77017
H	55	0.23708	0.00000	0.76185	0.00107	0.76292
H	56	0.24862	0.00000	0.75038	0.00100	0.75138

===

Table S6. Coordinates parameters for $[RuNOTSP]^+$ cation:

Center Number	Coordinates (Angstroms) X	Y	Z
1	1.800256	-2.653295	0.835584
2	2.893118	-3.157083	1.565152
3	1.860150	-2.786274	-0.548880
4	2.881345	-3.071078	2.644299
5	3.964014	-3.757180	0.940776
6	2.937139	-3.404952	-1.187675
7	4.787776	-4.136447	1.534594
8	2.937666	-3.504169	-2.267066
9	3.993274	-3.887143	-0.448139
10	4.830893	-4.365133	-0.938440
11	0.515831	-2.195527	-1.587563
12	0.489253	-1.916374	1.746906
13	-3.282282	0.868681	-0.935629
14	-4.110925	1.677137	-1.733916
15	-3.329386	1.088795	0.437883
16	-4.100788	1.533174	-2.806949
17	-4.931542	2.636117	-1.181255
18	-4.167869	2.049217	1.006548
19	-5.559134	3.241387	-1.825291
20	-4.189706	2.178050	2.082537
21	-4.965744	2.829617	0.199870
22	-5.615199	3.578055	0.634245
23	-2.336218	0.084601	1.551867
24	-2.285447	-0.336401	-1.743957
25	-1.088740	-1.322752	0.015916
26	0.155589	0.379788	-0.074956
27	0.055442	1.395189	0.834838
28	1.103415	0.516596	-1.051110
29	0.849587	2.511084	0.802690
30	1.942599	1.596911	-1.132642
31	1.844590	2.661100	-0.198584
32	0.707765	3.264498	1.557531
33	2.668575	1.624488	-1.926175
34	-2.269047	-3.713560	0.975794
35	-1.896245	-2.721741	0.404103
36	1.165947	-0.580691	-2.121404
37	2.189631	-0.708061	-2.467582
38	0.557761	-0.271647	-2.972864
39	-0.972544	1.218912	1.958914
40	-0.471492	0.795671	2.830725
41	-1.385868	2.184405	2.243600
42	2.646660	3.782657	-0.275302
43	2.554731	4.864018	0.737635
44	2.983000	5.758437	0.286272
45	1.503921	5.079891	0.933606
46	3.292255	4.555971	2.061151

47	4.356678	4.436307	1.889498
48	2.913262	3.649660	2.521920
49	3.146086	5.381861	2.750824
50	3.705319	3.901180	-1.309185
51	3.304720	3.571500	-2.268401
52	3.933693	4.961850	-1.408965
53	5.002783	3.127996	-0.978477
54	4.806273	2.070174	-0.838127
55	5.462407	3.515934	-0.075683
56	5.704997	3.243610	-1.798625
